# Predictors of male sexual dysfunction in post traumatic stress disorder

**DOI:** 10.1192/j.eurpsy.2021.1471

**Published:** 2021-08-13

**Authors:** R. Maalej, G. Hamdi, D. Felfel, H. Ben Ammar, A. Maamri, Y. El Kissi, H. Zalila

**Affiliations:** 1 Psychiatry Department, Razi, manouba, Tunisia; 2 Psychiatry, Tunisian Society of Clinical Sexology, TUNIS, Tunisia

**Keywords:** ptsd, Sexual Dysfunction

## Abstract

**Introduction:**

Post-traumatic stress disorder affects emotional, social and professional functioning. It can also affect physical and sexual health.

**Objectives:**

The aim of this study is to write down the prevalence of sexual dysfunction in men with PTSD and to economize on potential predictors of sexual dysfunction.

**Methods:**

A total of 30 male patients with PTSD were included in this study. We collected socio-demographic and clinical data and we used Post-Traumatic Stress Disorder (PCLS) and International Erectile Function Index (IIEF15) scales.

**Results:**

The mean PTSD severity score was 65.43 ± 2.95. The mean score for revitalization, avoidance, cognitive and mood alteration, and hypervigilance were 15.80 ± 1.44, respectively; 8 ± 0; 24.07 ± 1.20 and 17.57 ± 2.95. The mean IIEF-15 score was 51.16 ± 6.82. The mean sub-scores were 3.93 ± 0.52 for sexual desire; 18.80 ± 5.68 for erectile function; 8.93 ± 8.97 for orgasmic function; 5.13 ± 1.10 for satisfaction with intercourses and 4.13 ± 1.16 for overall satisfaction. The IIEF15-EF score was negatively correlated with the presence of a personal medical history (p = 0.02) and the impairment cognitions and mood score (p = 0.023). The IIEF-OF score was significantly associated with reviviscence, hypervigilance, cognition and mood alterations (p = 0.015; 0.041; 0.045). The IIEF-15 SD score was negatively correlated with altered cognition and mood (p = 0.007).

**Conclusions:**

Our study focused on the importance of assessing sexual function in men followed for PTSD and helps to understand the association of PTSD with different types of sexual dysfunction.

